# Just Another Level? Comparing Quantitative Patterns of Global Expansion of School and Higher Education Attainment

**DOI:** 10.1007/s13524-019-00775-5

**Published:** 2019-04-18

**Authors:** Bilal Barakat, Robin Shields

**Affiliations:** 1 Vienna Institute of Demography (VID), Austrian Academy of Sciences, Welthandelsplatz 2, 1020 Vienna, Austria; 2 School of Management, University of Bath, Bath, UK

**Keywords:** Educational attainment, Higher education, Educational expansion, Economic sociology

## Abstract

The expansion of higher education enrollment and attainment is a key uncertainty in the education profile of future populations. Many studies have examined cross-national determinants of higher education expansion as well the understanding of expansion through the relationship between higher education and the labor market. Early work established a typology for levels of enrollment, but recent empirical studies on the global growth of higher education attainment are scarce, and available projections resort to imposing ad hoc limits on future expansion. This study addresses this gap by comparing the trajectories of higher education expansion with those experienced at other levels on their course to universal or near-universal access. We demonstrate that a population-level model of expansion toward universal access fits higher education as well as lower levels of education (i.e., primary and secondary education). In other words, that there is no *prima facie* evidence of a ceiling in higher education enrollment that would indicate saturation significantly below 100 % participation. Claims that are premised on such a ceiling should therefore consider empirical evidence for this assumption in their analysis. These findings contribute to discussions on higher education expansion as well as studies of higher education and the labor market.

**Electronic supplementary material:**

The online version of this article (10.1007/s13524-019-00775-5) contains supplementary material, which is available to authorized users.

## Introduction

Demographic behaviors and experiences concerning fertility, health, mortality, and migration often vary considerably between individuals with different levels of education. For key characteristics, including life expectancy, such differences are observed even between the highest levels of formal educational attainment and academic distinction (Winkler-Dworak 2008). Although more education often comes hand in hand with other advantages, such as a higher income, the case for a direct effect of education is strengthened by findings that educational interventions result in measurable physiological changes (e.g., Skeide et al. 2017). Assessing the past and future implications of population aging for crucial issues, such as the burden of functional limitations and dementia, depends on the educational composition of the population (Crimmins et al. 2018; Freedman and Martin 1999; Martin et al. 2010), including its higher education attainment.

Most research on the expansion of education has focused on basic education through schooling. Spurred by global policy initiatives and civil society organizations, these studies have examined determinants of enrollment within a national development framework and theorized the accompanying social change (Boli et al. 1985; Hannum and Buchmann 2005; Levinson and Holland 1996; Meyer et al. 1992). The literature on higher education has also devoted considerable attention to the expansion of enrollments (Marginson 2016a; Schofer and Meyer 2005; Trow 1973), although generally without the attendant predisposition to truly universal attainment, in which *every* young person participates in higher education, as a normative goal. Indeed, a significant concern in research is whether attainment may reach levels that are undesirable (i.e., overqualification).

This study uses large-scale, cross-national demographic data to analyze trajectories of higher education expansion worldwide, understood to encompass both tertiary and nontertiary postsecondary education^1^ (such as nonacademic postsecondary trade schools and other courses falling under Level 4 of the International Standard Classification of Education (ISCED)). We argue that much research and policy has implicitly assumed that the trajectories of participation in higher education are fundamentally different from other levels of education, particularly with respect to ultimate ceilings. Our analysis therefore compares the functional form of higher education expansion in enrollments across nations with that observed in the expansion of primary and basic education to universal access. Although higher education differs from primary and secondary schooling in many ways, we empirically examine whether it follows a similar pattern of expansion or, unlike primary and secondary schooling, shows signs of reaching a ceiling at some significantly lower level. For researchers and policy analysts investigating education projections at the population level or feeding into population projections, the question is simply unavoidable. The ad hoc approach of artificially limiting higher education attainment to 90 % of a cohort (Lutz et al. 2014; Vincent-Lancrin 2008) is clearly unsatisfactory.

Our findings indicate that the expansion of higher education is remarkably similar to lower levels of schooling, merely at a slower average pace. In particular, we find that a model using the same functional form—a sigmoidal growth curve from zero to universal participation—fits the average expansion patterns at all levels equally well. In addition, the variation of individual countries around their average trend is similar, and so is the amount of variation between countries. The main difference between the levels of education is that the mean rate of expansion is slower for higher education.

## Theoretical Perspectives on Higher Education Attainment

Although absolute limits to expansion have seldom been discussed directly in the literature on higher education, much research has approached the topic indirectly, either by debating whether such limits have already been reached (i.e., overqualification) or by presenting theoretical arguments that entail expectations for the long-term trajectory of enrollment in higher education. We begin by discussing higher education expansion as it has been directly addressed in existing research literature and policy. We then analyze two broad theoretical perspectives to generate expectations for long-term trends in higher education enrollment. The first—*political economy approaches*—discusses higher education in terms of skills supply and labor market demand in the context of a policy and regulatory framework. The second—*institutional approaches*—views changing social norms around education as the driving force for expansion.

### Higher Education Expansion

Trow’s (1973) seminal study of higher education provides a framework for considering the expansion of higher education systems from elite (less than 15 % enrollment) to mass (16 % to 50 % enrollment) to universal access (greater than 50 % enrollment). However, the classification of 50 % enrollment as universal differs significantly from standards at other levels of education—specifically, the 100 % net enrollment target for universal primary education (UNESCO Institute for Statistics 2009; United Nations General Assembly 2000). To disambiguate, we use the term *truly universal* to refer to universal primary education. Foundational literature has established some expectation that in the long run, the expansion of higher education is likely to differ from other levels of education. However, Trow (1973:7) also noted the importance of changing social norms as a driving force in higher education expansion, stating that “when the proportion of the whole population comes to be about 50 %, and in certain sectors of the society it is then of course much higher, attendance in higher education is increasingly seen as an obligation.” Trow’s typology therefore leaves open the possibility for even higher levels of enrollment, despite establishing 50 % as sufficiently universal.

Upper plausible limits to higher education attainment are also implied in national and international policy documents. The European Union established a target attainment of 40 % (European Commission 2010:3), with other national targets ranging from 20 % to 60 % (Blair 1999; Bradley et al. 2008; Government of China 2010; White House, Office of the Press Secretary 2009). Similarly, international declarations on higher education have not put forward access goals in the same terms as used in basic education (i.e., the targets of truly universal access in Education for All), instead focusing more on equitable allocation of the existing enrollment capacity. Among international organizations, the OECD appears to be the most bullish, with Andreas Schleicher arguing, “We are moving to a world where tertiary education is where secondary education was 100 years ago” (quoted in Sharma 2015). Although his statement may be interpreted as strictly descriptive, the OECD’s treatment of intergenerational educational mobility carries more normative overtones, given that only ever-continuing expansion can reduce downward mobility at the same time as reducing the dependence of children’s education on that of their parents, both of which are considered undesirable (OECD 2015). The normative case for a universal *entitlement* to higher education has been made explicit and elaborated as a logical implication of the notions of Education For All and Lifelong Learning (McCowan 2012).

The fact that many policy documents implicitly assume an upper limit to higher education expansion suggests that they see the reasons for this limit as obvious. Indeed, it is easy to see some common sense arguments from a policy perspective: higher education participation typically occurs beyond the age of legal majority, and it therefore has no prospect of becoming compulsory.

In summary, foundational research on higher education expansion and policy are somewhat ambiguous on limits to expansion. Both have tended to establish realistic near-term limits (i.e., 20 % to 60 %) while leaving the possibilities for long-term expansion open.

### Political Economy

Early work on human capital theory (Becker 1964; Mincer 1958; Schultz 1981) established economic returns to education, providing a rationale for both individual and national investment in higher education. More recently, research has focused on the *skills premium* (Autor 2014), usually operationalized as the average increase in earnings for university graduates versus nongraduates.

In contrast, the literature on overeducation has examined limits to higher education expansion by seeking to establish the extent to which university graduates take jobs that do not seem to require a degree. Perhaps because of the challenges of operationalizing overeducation, evidence from the literature is ambiguous. One meta-analysis concluded that “the impacts of over-education are likely to be non-trivial” (McGuinness 2006:387), and other studies noted that findings vary across national contexts (Barone and Ortiz 2011; Reisel 2013) and reported increasing variability in the premium (Figueiredo et al. 2013; Green and Zhu 2010).

Perspectives such as endogenous growth theory and skill-biased technical change emphasize the elasticity of the demand for skilled labor, arguing that higher levels of human capital can increase the demand for it (Barro and Sala-i-Martin 2004; Lucas 1988). Holding a similar view, proponents of skill-biased technical change have argued that changes in technology tend to favor skilled over unskilled labor in the job market (Autor et al. 1998). Thus, higher levels of technology will lead to a higher demand for skilled labor in *knowledge societies* (Nowotny et al. 2001) and increase the incentive for individuals to complete higher education. Although the expansion of higher education associated with technological change could have limits, this literature raises the possibility that limited demand for skills need not cap higher education expansion. In any case, even if conceived as a market responsive to price signals, the limited transparency and information asymmetries in higher education (Weisbrod et al. 2008) imply that the sector may face “difficulties in correcting supply and demand mismatches” (Figueiredo et al. 2015:3) and will continue to produce graduates over and beyond the needs of the labor market. Indeed, expansion of higher education does not even rely on the political will to fund it. Strikingly, higher education enrollment rates are actually negatively associated with the share of the education budget for higher education (Bergh and Fink 2008; Mohamed 2008).

The *credentialing perspective* claims that rather than meeting an unfulfilled demand for skilled labor, higher education is used to exclude individuals from occupations and perpetuate class advantage (Brown 1995; Collins 1979). The result is *credential inflation*, a “cycle of rising educational attainment and rising occupational requirements” that threatens to continue “until janitors need PhDs” (Collins 2002:25–29). However, Collins predicted an adjustment of the labor market, noting historical declines in school enrollments in Spain, Germany, and France (Collins 2002:30). To the extent that education is used to differentiate individuals, *credentialist theory* expects some variation in attainment and therefore some limits to the expansion of higher education. However, Green (1980) offered a general argument that once a given credential becomes sufficiently common and therefore is normalized, the credential ceases to command a premium, and instead its absence becomes associated with the stigmatization of negative selection (Solga 2002). Social class reproduction through higher education does not necessarily rely on maintaining a gap in formal attainment (Marginson 2016b) because such differentiation can still occur through institutional status (Parry 2011), higher levels of degree education (i.e., master’s and doctorate degrees), quality (Alphen 2012), or subject choice (Dias Lopes 2016).

### Social Norms and Institutional Theory

*Institutional perspectives* emphasize the importance of shared social norms and rationalizations in shaping human behavior, suggesting that they are stronger determinants of social behaviors than economic circumstances or rational choice (Meyer and Rowan 1977). In the literature on institutional theory, the rapid expansion of higher education is facilitated by the strongly engrained model of the university and its rationalization through universal principles such as truth, knowledge, autonomy, and excellence (Frank and Meyer 2007; Meyer et al. 1977; Ramirez 2010; Ramirez and Tiplic 2014). Thus, Schofer and Meyer (2005:917) found that membership in international organizations—which they viewed as conduits of “a common universalistic culture”—is a better predictor of higher education expansion than the functional need for skilled labor.

Baker (2009, 2011) also sought to explain the why the *diploma disease*—widespread overeducation among university graduates (Dore 1976)—has never materialized in most countries. According to Baker (2009:166), “as more waves of educated individuals flooded the workplace, . . . there were sustained shifts towards jobs with more managerial, communicative components, that yielded a spread of a kind of mass professionalisation of work.” This created new social norms that established formal education as a legitimate means of differentiation and selection. From this perspective, the rapid increase in higher education enrollment is better explained by these shared norms than by a functional shortage of skilled work. For the same reason, there is little concern that changes in labor market will limit the expansion of higher education enrollment if these underlying norms and values continue to hold currency.

In addition, the institutional perspective provides an explanation for the observation “that some of the wealthiest nations can have low tertiary graduation rates” (Andres and Pechar 2013:1), including in particular the German-speaking countries. Crucially, although relatively low past and present tertiary participation in these countries can be explained in terms of institutional models of capitalist labor markets (i.e., the “varieties of capitalism” of Hall and Soskice (2001)), this does not rule out significant tertiary expansion in the future, given that “it is not clear whether the strong emphasis on upper secondary vocational training that unquestionably contributed to their economic success during the Fordist era of capitalist development is still a comparative advantage in an increasingly knowledge-based economy” (Andres and Pechar 2013:12).

In considering the long-term trajectory of higher education enrollments, the *institutionalist approach* provides a challenge to the assumption that expansion is linked to or is explained by economic and functional concerns, which it regards as common myths. Instead, this perspective generally expects that higher education will expand where norms about higher education are shared, even discussing “the possibility of universal higher education” (Schofer and Meyer 2005:898)—in our sense of truly universal—as discourse on higher education is standardized at the global level.

### Hybrid or Intermediary Theories

Other theories incorporate elements of both political economy and institutional perspectives. This *hybrid approach* is best illustrated by Clark’s (1960) foundational work on the “cooling-out function” of higher education. According to Clark, contemporary society embeds a mismatch or contradiction between the “encouragement to achieve and the realities of limited opportunity” (Clark 1960:659). In other words, the normative emphasis on knowledge, education, and achievement are limited to some extent by the realities of the labor market. The role of higher education is then to reconcile this contradiction through a process of soft denial that includes establishing alternative means of success and a gradual process of disengagement from students’ original aspirations.

Literature on screening and signaling takes a different approach to the labor market, asserting that in a competitive environment with limited information, employers rely on certain signals from applicants to screen the supply of labor (Bills 2003; Spence 1973; Stiglitz 1975). From this perspective, higher education signals increased levels of ability or motivation to employers, which influences hiring behavior. The signaling perspective implies continued expansion of higher education insofar as it continues to hold signaling value, which depends upon both employer’s normative preferences and the prevalence of attainment among job-seekers. In any case, some nonmonetary benefits of higher education, such as improved health (independently of income) (McMahon 2009), are noncompetitive and may therefore serve as justification for continued expansion independently of the labor market demand for graduates.

### Summary

The preceding review demonstrates a lively disagreement simply regarding the expectations for what kind of higher education expansion dynamics will, in fact, prevail, even leaving aside normative debates about their relative desirability. Some have argued that education expands to meet labor force needs that are limited, and others have suggested that these needs in turn are potentially unlimited. By contrast, educational expansion may represent a competitive inflation, with disagreement about whether this is self-correcting or self-reinforcing. Finally, its social value in society may justify expansion not limited by concrete need. The long-term trajectory of higher education is thus an empirical question, one which we propose to investigate with an expansive data set and unique methodological approach.

**Fig. 1:**
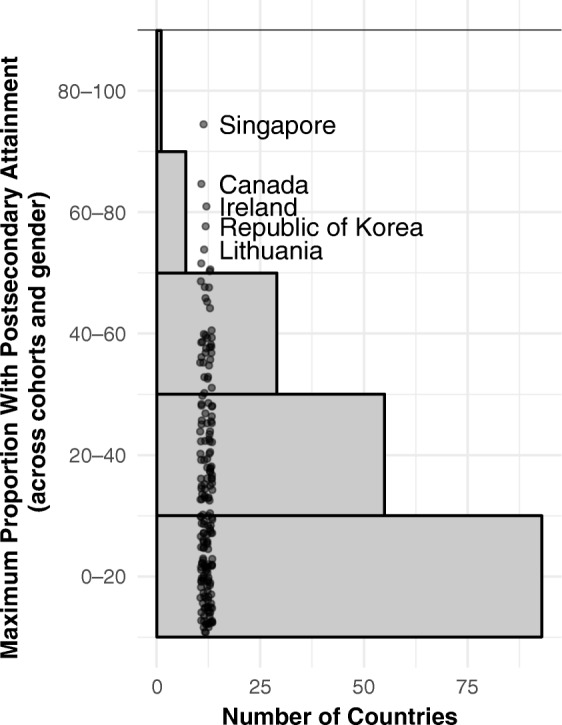
Distribution of maximum proportion with postsecondary attainment in any five-year cohort, by gender, in reference years 2010–2015. The top five countries are labeled.

The question is timely, now that higher education attainment has reached unprecedented levels in frontrunner countries. Figure 1 provides a sense of the global status quo with respect to postsecondary attainment. In roughly one-half of all countries, fewer than one in five achieve postsecondary attainment. Yet in a significant number of cases, already around one-half of young people and two-thirds or even more in these frontrunner countries do so. Notably, although these are all high-income economies, they represent a variety of population sizes, cultures, and welfare systems.

## Methods and Data

Our approach is to model expansion at all levels of education (i.e., primary, lower-secondary, upper-secondary, and postsecondary) using the same statistical specification. We then compare across education levels the structure of the residuals and the distribution of the country-specific parameters that capture the rates of expansion. This comparison indicates the extent to which higher education expansion has the same functional form as that of lower levels and merely differs in terms of the specific values of the parameters. In other words, we investigate whether the observed pattern of higher education expansion is consistent or inconsistent with a trajectory that, like those of the lower education levels, has no ceiling below 100 %.

### Data

The empirical historic expansion patterns are estimated on a recent update (Lutz et al. 2018) to the set of global reconstructed time series of completed educational attainment (Lutz et al. 2014). These are disaggregated by country, year in the range 1950–2015, gender, five-year age groups, and six education levels (none, incomplete primary, primary, lower secondary, upper secondary, and postsecondary). The latter is an aggregate category that includes, but is explicitly not limited to, tertiary education. These time series are reconstructed from the most recent available large-scale, cross-sectional baseline data. In most cases, censuses (either complete information obtained directly from national statistical offices, or public use subsamples) are used, with remaining gaps filled with regional or international large-scale household surveys with tens of thousands of households, such as the Demographic and Health Surveys (DHS). The median baseline year is around 2010 in all world regions except in Northern Africa (2005) and Central Asia (2005), although in exceptional cases, the latest usable data are 15–20 years old. In the present exercise, 185 countries are included, covering a vast majority of the global population, and most exclusions are small (island) states.

Because the baseline data build on censuses and large-scale surveys, a minimum level of security and state capacity is normally required for countries to be included; thus, failed states and countries suffering from violent conflict are underrepresented in the data. Assuming that these countries also exhibit below-average rates of educational expansion, overall and regional trends are biased upward to some extent in their absence. However, there is no reason to expect the missing countries to affect the results concerning the degree of similarity between higher education and lower levels of schooling.

The consolidated and harmonized baseline data are back-projected along cohort lines, accounting for educational mortality differences. As an illustration of the basic principle, and ignoring said mortality selection, the share of 50-year-olds with at least upper-secondary education in the year 2000 informs us of the likely share of *40*-year-olds in 1990. Where possible, these back-projections are validated against historic data sources. Crucially for present purposes, the reconstruction of the attainment time series requires only assumed educational mortality differences and imposes no assumptions regarding the historic trajectories of attainment expansion. In other words, although the reconstructed trajectories are partly model-based, our analysis of their form does not simply recover assumptions that went into the reconstruction.

Typical durations and graduation ages for different attainment levels unfortunately do not line up with this five-year grid. To ensure that most late attainment is captured, completed primary attainment is observed at ages 15–19; completed lower secondary, at ages 20–24; and completed upper secondary and higher education, by ages 25–29. The latter is likely to underestimate the amount of higher education attainment somewhat because those who complete higher education after age 29 would not be measured, but setting an even higher reference age for completion would come at the cost of a greater time lag and less current observational data.

### Model

**Fig. 2:**
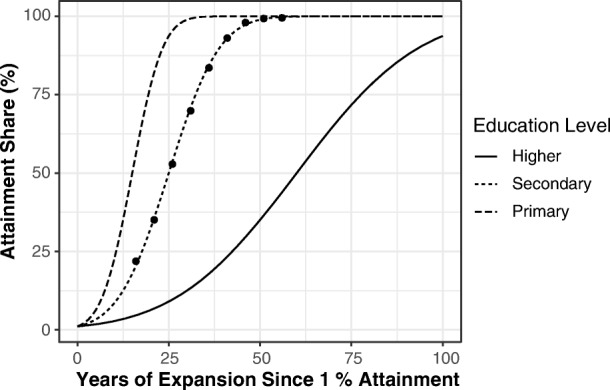
Illustrative stylized expansion trajectories with a shared functional form but distinct expansion rate parameters. Dots represent female lower secondary attainment in the Republic of Korea.

We model the proportion of individuals aged 25–29 who have completed a given level of education as following approximately a sigmoidal (i.e., S-shaped) curve from 0 % to 100 %. Figure 2 illustrates several features of the analysis. First, it shows what is meant by a sigmoidal expansion trajectory. Second, it shows what it means for different trajectories to share this same shape but to differ in terms of rate of expansion. Third, by overlaying a select empirical example—namely, female secondary enrollment in the Republic of Korea—it shows that the specific functional form is a reasonable idealized trajectory.

The sigmoid pattern provides the context for defining a constant rate of expansion. What would maintaining a constant rate of expansion look like for a country that moves from 90 % in 2000 to 96 % in 2005? To answer this question, we perform the analysis on a transformed scale that turns the sigmoidal curve into an unbounded linear trajectory. The validity of the transformation—specifically, a probit transformation—is borne out by the results, which show that on the transformed scale, the residuals closely follow a random normal distribution around this idealized constant expansion path. Hereafter, all references to expansion rate refer to the linear transformed scale, not the original percentage scale.

On this probit-transformed scale, the attainment trajectories continue from the previously reached position at each step but potentially retrace part of the previous period’s move. Formally,

$$ {y}_{c,t,g}=\Phi \left({\uplambda}_{c,t,g}+{\epsilon}_{c,t,g}\right) $$

$$ {\uplambda}_{c,t,g}={\uplambda}_{c,t-1,g}+{\uptau}_{c,g}+{u}_{c,t,g}-\uptheta {u}_{c,t-1,g}, $$

where *y*_*c*,*t*,*g*_ is the share between 0 and 1 reaching a given attainment level in country *c* at time *t* among gender *g*, λ_*c*, *t*, *g*_ is the predictor of *y* at the linearized scale, Φ is the cumulative density function of the standard normal distribution, and the **u** are the random disturbances to attainment. The **ϵ** account for exogenous errors at the level of data, rather than in the underlying educational process. The scale of the **ϵ** is set to an order-of-magnitude smaller than that of the **u***a priori*, and it is the latter that are identified as the *residuals* in the analyses that follows. Parameter θ captures the extent to which deviations from the central expected trajectory persist over time. The main parameters of interest are the country- and gender-specific parameters, τ, which represent the expansion rate. Additional complexity is layered over this basic model. The extent to which male and female attainment rates converge is estimated endogenously, for instance. The model is estimated within a Bayesian framework (with vague prior, see the online appendix) (Gelman and Hill 2007; Gill 2006).

This model is deliberately purely structural: no explanatory covariates are included because none are available for sufficiently many countries and years. In any case, their omission reflects our intention of understanding the de facto expansion dynamics, rather than an isolated *ceteris paribus* effect. Indeed, in terms of informing our future expectations, a parameter estimate for the contribution of GDP, say, would provide little to no benefit because projections of economic growth several decades into the future are on no firmer footing than projections of educational attainment itself.

## Results

### Distribution of Residuals

The first step in the analysis of the model must be whether it provides an appropriate fit to the data. For our purposes, a single summary measure of the residual distribution will not do. Instead, we require a more careful examination of their actual distribution. Specifically, we investigate whether the residuals (1) follow a random, normal distribution at different stages of expansion that (2) is similar across education levels. If this can be shown, then we can conclude that the functional form encoded in the model specification does indeed capture the essential shape of the underlying long-term trajectory, and it does so equally well for all education levels.

**Fig. 3:**
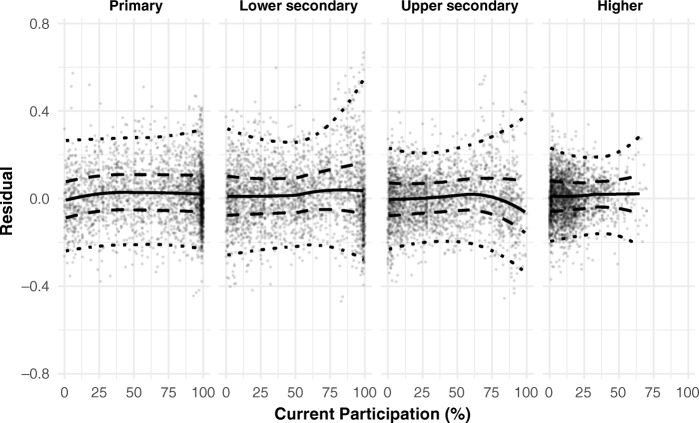
Residuals by education level and stage of expansion. Smoothed quantiles shown: median (solid line), 0.25 and 0.75 (dashed), and 0.025 and 0.975 (dotted). Quantiles above 65 % participation for higher education are omitted because of the small number of observations.

The residuals at each education level, sorted by current attainment share, are shown in Fig. 3, together with smoothed intervals containing 50 % and 95 % of the residuals, respectively. The 95 % boundaries are intrinsically more variable, but they clearly exhibit a fairly consistent magnitude across education levels. The more robustly estimated inner bounds are remarkably similar in magnitude at all levels. Moreover, both the quantiles and the smoothed medians are essentially constant across different stages of expansion. This confirms that the sigmoid-shaped trajectories implied by the probit model can indeed be thought of as representing expansion at a constant rate. If this were not the case—for example, if the natural leveling-off of expansion instead occurred later and more abruptly than implied by the probit model—then the residuals would be systematically biased upward at later stages of expansion.

**Fig. 4:**
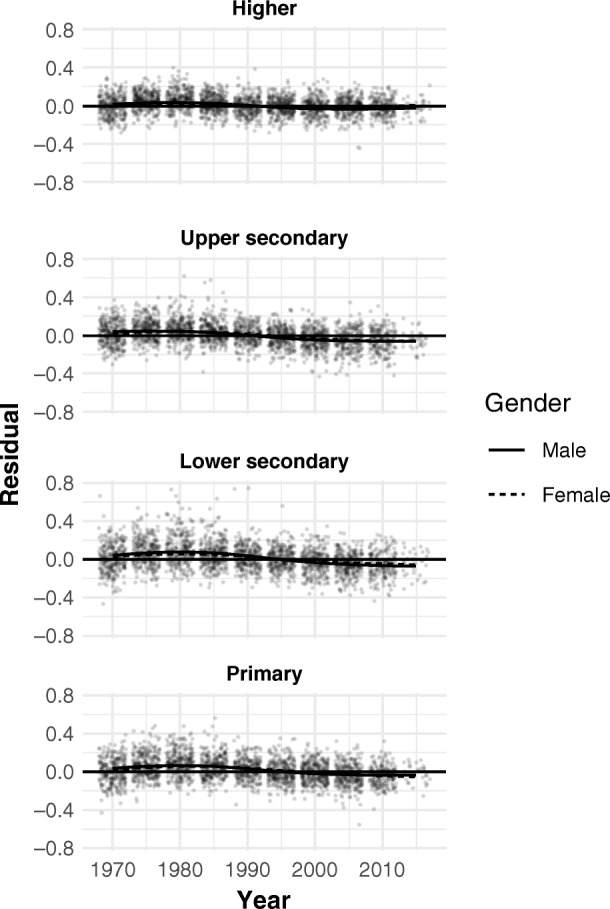
Residuals by education level and period. Smoothed means by gender are shown. Year corresponds to the cohort aged 30–34 at the time.

The model does not impose convergence in attainment between females and males, but to the extent that the gender-specific rates are pulled toward their joint average, the strength of this effect is estimated. As is evident in Fig. 4, there are no noteworthy residual gender differences in the overall model fit. In other words, our conclusions hold equally for higher education attainment growth among males and females.

Figure 4 also shows an absence of significant period effects. Specifically, even fully nonparametric period effects account for less than 10 % of residual variation (across all education levels). For a more interpretable model with region-specific linear time trends, the proportion is only around 7.5 %. In other words, the effect of different eras of universally slower or faster educational expansion is at best marginal. Moreover, such a weak temporal pattern may well represent a statistical artifact. It would arise, for example, if the fit of the probit model to the true curvature varied along different parts of the curve, and the bulk of countries move from one stage to another. Similarly, suppose the accuracy of the mortality differences by education that enter the back-projection differed systematically between cohorts. Then the earlier values in the attainment time series would be systematically over- or underestimated relative to more recent values, inducing a spurious time trend.

In summary, the magnitude of residual temporal patterns is too small and uncertain to call into question the present conclusions based on the time-invariant country trends that are almost an order of magnitude larger, especially because there is no clear way in which the residual temporal pattern (such as there is) for higher education differs from other levels.

### Distribution of Expansion Rates

**Fig. 5:**
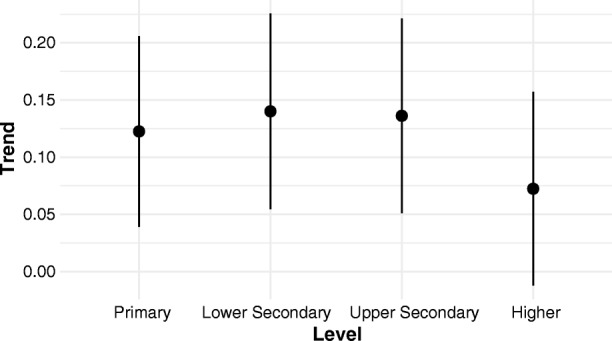
Distribution of country- and gender-specific expansion rate parameters by level. Mean (dot) and +/– 2 standard deviations (bar) are shown.

Although the preceding analysis of the residuals already establishes that higher education follows the same pattern of expansion as lower levels of schooling, the similarities go even further. The distributions of country-specific expansion rates at different levels are summarized in Fig. 5. As expected, the *mean* rate of expansion is indeed generally slower, the higher the level of education. The primary expansion rates appear to break this pattern. However, data points that are very close to 0 % or 100 % must be omitted from the estimation because these values correspond to negative infinity and positive infinity, respectively, on the transformed scale. Accordingly, countries with near-universal primary schooling throughout the observed period are not represented among these primary expansion rates; conversely, countries with slow primary expansion are overrepresented. Remarkably, the distributions of expansion rates at different education levels display considerable overlap. In other words, relatively speaking, a considerable number of countries expand higher education at a faster rate than other countries expanded secondary or even primary schooling.

The spread of higher education expansion rates is highly consistent with other education levels. This means that uniformly speeding the expansion of higher education would make the global ensemble of higher education expansion trajectories practically indistinguishable from those of upper-secondary or (with a different speed-up factor) lower-secondary or primary schooling.

### Correlation Structure

There is no limit to other explicit and implicit structural model characteristics that could be compared across education levels. Among the possible secondary analyses, the correlations of expansion rates and residuals between levels are of particular interest and are shown in Table 1. The former shows the extent to which countries where upper-secondary attainment is expanding rapidly/slowly in general are also likely to have rapid/slow expansion trajectories at lower-secondary or higher education, for instance. The latter shows the extent to which countries experience short-term shocks or booms in their expansion trajectories that affect multiple levels at once, or whether each level experiences its idiosyncratic random deviations from the expected mean trajectory.

**Table 1:** Cross-level correlations between expansion rates (upper triangle) and between residuals (lower triangle)

	Higher	Upper Secondary	Lower Secondary	Primary
Higher	––	.68	.57	.39
Upper Secondary	.33	––	.64	.42
Lower Secondary	.24	.46	––	.60
Primary	.22	.32	.36	––

The moderately large cross-level correlations between long-term expansion rates (Table 1, upper triangle) show that, unsurprisingly, countries have a tendency to be leading or lagging in educational expansion overall. Nevertheless, in quite a few cases, one level is expanding relatively rapidly but another is expanding relatively slowly compared with other countries. What stands out is that the expansion rate of higher education is very highly correlated with that of upper-secondary schooling—more so, in fact, than upper-secondary expansion correlates with lower-secondary expansion. This finding is far from obvious *a priori*: the two secondary levels often (although certainly not always) share physical facilities and even teaching staff. In principle, such a high correlation would be expected if higher education were constrained by the availability of eligible entrants completing upper-secondary school, but in practice, the expansion of higher education is often so far behind that this is rarely a binding constraint. That expansion at the two upper levels does correlate strongly undermines the notion that the transition between them represents a qualitatively distinct kind of break in educational progression.

With respect to the cross-level correlations between residuals (Table 1, lower triangle), it is evident that there is nothing remarkable about higher education, which seamlessly fits with the correlation pattern prevalent among lower levels. That pattern itself predictably shows less correlation between levels further apart. The overall level of correlation is moderate. Although there are certainly shocks that affect the education system as a whole, evidently many positive and negative disturbances affect specific levels. In addition, positive correlation from sector-wide shocks may be partly offset by negative correlation induced by shifting spending priorities, for example, where one level is supported at the expense of another.

## Discussion and Conclusion

This study contributes the first comparison of higher education expansion with the trajectories of primary and secondary education on their course toward truly universal access. On the basis of our results, we conclude that the quantitative pattern of the expansion of higher education attainment is the same as that of lower levels of schooling but is slower. One implication is that this level of analysis produces no evidence of higher education expansion topping out markedly below truly universal participation. Instead, we find much support for the notion that higher education is the next secondary education—in a well-defined technical sense in terms of the quantitative pattern of the expansion of formal attainment. Patterns of expansion in the gross enrollment ratio in tertiary education further support this conclusion (see the online appendix, section B).

This conclusion is empirical, not normative. Under the assumption that higher education will become universal only if this would be beneficial, the normative and empirical perspectives could not be disentangled. However, as the literature review shows, there are arguments for expecting continued expansion regardless of whether it is beneficial at the individual or aggregate level. Our own position is that the possibility that a sustained trend toward universal higher education will manifest either way must be taken seriously. Over the course of the twentieth century, we have moved from asking who can benefit from 12 years of schooling to asking how 12 years of schooling can be designed to benefit everyone. We argue that in the interest of both equity and efficiency, we have to start grappling with the same question with regard to higher education.

As discussed earlier, literature from a variety of theoretical perspectives expresses diverging views on the prospect of (close to) truly universal access to higher education. For example, proponents of skill-biased technical change, endogenous growth, and institutional theories have argued that higher education could reach truly universal levels either because of changes in the labor market or shifting social norms around higher education as an institution. These perspectives gain some support from present findings, which suggest that such a pattern of expansion is entirely consistent with current trends and historical patterns of enrollment at other levels. Our findings are harder to reconcile with respect to other perspectives. The literature on overqualification, credentialism, and screening/signaling suggests the expectation of some upper limits on participation, but although our results suggest that these upper limits may well be reached in the future or in certain contexts, we find no evidence of widespread or systematic ceilings on attainment to date.

Because higher education is evidently not intrinsically *sui generis* in terms of its expansion trajectory, if we do *not* believe that higher education will become (close to) truly universal, we need to be more precise in specifying the point at which—and why—we would expect the expansion dynamic to undergo a fundamental change. This would provide an opportunity to study in greater detail how different inflationary or limiting factors dominate along different parts of the trajectory. The factors that *initiate* a phase of rapid higher education expansion may well be different from the factors that *sustain* it. Systematically structuring the study of the determinants of further higher education expansion by the level of participation already achieved would be prerequisite for a convincing argument as to why higher education is reacting similarly to inflationary pressures at low and medium levels of expansion but nevertheless should be expected to react differently to the forces crucial at the top. The opportunity to study this issue is available: if a ceiling to higher education expansion exists in the range of 60 % to 80 %, evidence of it will be clear within the next 20–30 years from a relatively large number of countries. It would be a Eurocentric fallacy to conceive of different levels of education as universalizing more or less sequentially and in distinct eras. In India, for example, the phases of rapid expansion at different levels are happening much more concurrently.

To interpret the implications, recall that expansion of attainment shares, as indicative of expanding participation rates, must not be confused with expansion in terms of absolute numbers. In particular, perceived massification of postsecondary and tertiary education in many Western European countries occurred at participation rates that—from today’s perspective—were still rather moderate because large Baby Boomer cohorts entered the relevant age group at the time. In general, perceptions of whether absolute growth has slowed, even from within the sector, are of limited relevance to the present question of where participation *rates* are headed.

Quantitative patterns of expansion similar to those at lower education levels obviously do not deny that higher education is very different from lower levels of education in other ways. Indeed, even the slower average pace of expansion *by itself* has substantive implications in relation to social dynamics—implications that differ from those of more rapid expansion at lower levels of schooling. For example, the length of a generation varies (over time or between social groups) by less than the difference in the pace of expansion between levels of education. This fact alone implies different intergenerational dynamics (Blossfeld et al. 2017): fast primary expansion is much more likely to be inequality-reducing, for example, because most of the new beneficiaries necessarily come from families in which the parents themselves did not complete primary schooling. By contrast, higher education expansion—*on account of its slower average pace*—leaves much more room for it to be largely monopolized by the children of more-educated households.

## Electronic supplementary material

ESM 1 (DOCX 175 kb)

## Footnotes

1 As shown in section A of the online appendix, combining both forms of higher education is not consequential for our findings.
