# Supplementary material for: Just Another Level? Comparing Quantitative Patterns of Global Expansion of School and Higher Education Attainment
Source: Demography. 2019 Apr 18;56(3):917–34. doi: 10.1007/s13524-019-00775-5 (PMC6592959; doi:10.1007/s13524-019-00775-5)
Supplement: Supplementary file 1 — (DOCX 175 kb) [file 13524_2019_775_MOESM1_ESM.docx]

**Just another level? Comparing quantitative patterns of global school and higher education expansion**

**Online Appendix**

## **Tertiary Versus Non-Tertiary Post-Secondary Attainment**

The above analyses cannot be performed separately for non-tertiary and tertiary types of post-secondary attainment, because this disaggregation is unavailable for a majority of countries. On the subset of countries for which the detailed categories can be distinguished in the cross-sectional data for the baseline year, a supplementary analysis shows that the split of overall post-secondary attainment between tertiary and non-tertiary lacks any meaningful systematic trend at the global level (Fig. A1). Over cohorts (a), there may have been a slight shift from tertiary towards non-tertiary post-secondary attainment. However, this marginal trend is an order of magnitude of smaller than the baseline level variation between countries. In any case, more salient for the present question is the relationship between the non-tertiary/tertiary balance and the stage of overall post-secondary expansion (b). From this perspective, there is even less evidence of a generalisable pattern. In other words, expanded mass participation at the post-secondary level is not necessarily, or even typically, concentrated at the lower, non-tertiary, end of post-secondary attainment, in a way that would change the implications of continued post-secondary expansion as discussed in the main text.

[Figure 6 here]

## **Trends in Tertiary Enrolment**

While data on tertiary enrolments are more timely, they offer little insight into ultimate attainment in real-life, dynamic settings. The only indicator available on a global basis is the Gross Enrollment Ratio (GER) in tertiary education. This is defined as total tertiary enrolments divided by the population in the five-year age group following the standard secondary school graduation age. As a proxy for cohort lifetime tertiary attainment, this is distorted by the fact that a degree may be attained in less than five years, and both by drop-out and study at later ages. The latter is very common in many countries, and perhaps increasingly so in the context of ‘lifelong learning’. Moreover, it is clear for demographers in particular that even at constant attainment, the tertiary GER will change in response to changes in the duration and timing of enrolment even within the five-year window. As a result of all these dynamics, a tertiary GER of 120% may well arise in a situation where far from all young people attain a tertiary degree, and a tertiary GER of 80%, say, in a situation where all do. In other words, trends in enrollment in principle cannot provide conclusive evidence for or against a ceiling in attainment. For what it is worth, the global (unweighted cross-country average) tertiary GER has been rising continuously (Fig. A2, solid line). This is also true for the subset of countries where it has already risen above 70% (dashed line) (both averages would show a slight drop for 2016, however this value is not actually comparable because the 2016 figure is still missing for a third of countries). In other words, at the global level, enrolment trends do not even provide face-value evidence in favour of a ceiling effect, and an analysis based fully on enrollments rather than attainment leads to similar conclusions (Marginson 2016a).

[Figure 7 here]

## **Bayesian Prior Distributions**

The Bayesian estimation framework requires the specification of prior distributions for all parameters. Here, vague priors are specified that only incorporate knowledge of the order-of-magnitude of various effects, as well as logical bounds, and do not drive the results.

The mean-reversion effect $\theta$ has a Beta(1.5, 1.5) prior in the interval (0, 1). The estimated gender convergence factor $\nu$ is level and country specific, with prior Beta(1, 5), to ensure a value in the interval (0, 1), strongly skewed towards smaller values. True initial levels are given conceptually uninformative ‘flat’ priors, but restricted to the interval (-4, 4) to ensure a proper posterior. The idiosyncratic shocks at the probit scale, i.e. the gender, level, year, and country specific epsilons, are i.i.d. draws from a Gaussian distribution with zero mean and standard error $\sigma_{\epsilon}$. The additional errors stem from a Gaussian N(0, 0.05) distribution. The (gender, level, and country specific) drift parameters have Gaussian priors centred on regional means (themselves drawn from a Gaussian N(0, 1) distribution), with standard error $\sigma_{trend}$. The hyper-priors on variance parameters $\sigma_{\epsilon}$ and $\sigma_{trend}$ are Gaussian with mean zero and variance 0.2.

Fig. A1 Tertiary/non-tertiary composition of post-secondary attainment on cross-sectional sub-sample (latest available year) for which detailed higher attainment data is available. Log-odds-ratios: higher values favour non-tertiary.

1. over age/cohort.
2. over overall level of post-secondary attainment

Fig. A2: Gross Tertiary Enrolment Ratio (Tertiary GER), all available countries. Solid line: unweighted global average. Dashed line: unweighted average among countries ever exceeding 70% (excluding volatile outliers). Data: World Development Indicators (WDI).
